# The use of methylprednisolone after third molar surgery. A systematic review and meta-analysis of randomized controlled trials

**DOI:** 10.4317/medoral.26842

**Published:** 2024-12-24

**Authors:** Hélio Libório, Tiago Borges, Miguel Pereira, William Ross, Juliana Campos Hasse Fernandes, Gustavo Vicentis Oliveira Fernandes, Bruno Leitão-Almeida

**Affiliations:** 1 MSc (HL, MP). Faculty of Dental Medicine, Universidade Católica Portuguesa, Viseu, Portugal; 2 PhD (TB, BL-A). Centre for Interdisciplinary Research in Health (CIIS), Universidade Católica Portuguesa, Viseu, Portugal; 3 DDS. Private Researcher, St. Louis, MO, U.S.A.; 4 PhD (GVOF), sDDS (WR). A. T. Still University, Missouri School of Dentistry and Oral Health, St. Louis, MO, U.S.A.

## Abstract

**Background:**

Extraction of impacted third molars is a standard procedure in dentistry. However, the postoperative inflammation and pain are undesired and uncomforTable. Methylprednisolone has emerged as a possible solution to improve outcomes. This systematic review aimed to evaluate methylprednisolone in the postoperative period of impacted third molars in relation to its efficacy in postoperative pain and edema, dosage regimens, administration forms, and adverse effects.

**Material and Methods:**

Following the PRISMA guidelines, the focus question was: “In patients who underwent impacted third molar extraction, what was the effect of methylprednisolone used postoperatively compared to non-use or the use of other medications within the same pharmacotherapeutic group to reduce inflammation and pain?” PubMed/MEDLINE and Scopus were consulted, and an additional manual was performed. The search included articles published in the last 10 years, with the language restriction to English. The articles were analyzed using the PRISMA principles, with pre-defined eligibility criteria. The data were extracted based on the general necessary information. The risk of bias for the RCTs included was performed using a revised Cochrane risk-of-bias tool for randomized trials, and a meta-analysis was performed.

**Results:**

Nine articles were included, and five were quantitatively analyzed. Evaluating the test and control groups (methylprednisolone versus controls), there was no significant heterogeneity for pain at 24 hours (*p*=0.15, I²=47%) and 7 days (*p*=0.15, I²=47%), with non-significant effects (*p*=0.85). In the inter-incisal reduction, there was homogeneity at 48 hours (*p*=0.96, I²=0%) and 7 days (*p*=0.37, I²=0%), with a greater reduction in the methylprednisolone group (*p*<0.01 and *p*<0.05, respectively).

**Conclusions:**

Methylprednisolone is efficient in safely treating patients after third molar extraction, reducing pain, edema, and trismus. It achieved better results for the inter-incisal level than dexamethasone; otherwise, dexamethasone is preferable in minimizing postoperative trismus, presenting superior potential in this specific clinical context.

** Key words:**Third molar, impacted teeth, methylprednisolone, corticosteroids, oral surgery.

## Introduction

Third molar extractions are typically a complex procedure that requires a significant amount of time, experience, and knowledge from the professional. The location of third molars is anatomically complex due to the innervation and vascularization of these teeth. Then, post-operative sequelae, such as edema, pain, and trismus, are expected to appear immediately after surgery. Following the procedure, the worst-case scenario could include: infection, dry socket, paresthesia, and fracture (1). Usually, the referral for extraction is based on these associated pathologies. In the case of asymptomatic third molars without pathology, it is possible to monitor the patient according to the risk-benefit ratio evaluation (2,3).

Pain, trismus, and swelling are the most common postoperative complications in this type of surgery. Therefore, understanding that it is an invasive procedure, the professional must provide the patients with the most appropriate pre-, intra-, and post-operative care (2). The complications are also associated with other factors, such as the degree of tooth impaction, patient's age and health status, surgeon's experience, smoking habits, use of contraceptives, and technique used (4). Thus, there are different ways of approaching the complications depending on the type of surgery and patient.

Corticosteroids are one of the most common medications given for post-operative complications. Corticosteroids are prescribed for various conditions and have a wide range of effects on the human body. They are synthetic analogs of natural steroid hormones produced by the adrenal cortex. Their function and objective is to reduce inflammation by suppressing the immune system (5), decreasing cellular permeability and capillary dilatation by inhibiting the production of vasoactive substances and diminishing the amount of cytokines (6). Furthermore, corticosteroids repress the generation of prostaglandins, obtaining an analgesic effect (7). Corticosteroids can be divided into two groups: glucocorticoids and mineralocorticoids. Glucocorticoids have anti-inflammatory properties with minimal or no influence on the fluid or electrolyte balance (8). Regarding biodistribution, corticosteroids are immediately absorbed in the gastrointestinal tract, where they vigorously bind to proteins, undergoing hepatic metabolism and renal excretion. These drugs are available in oral, intramuscular, intravenous, intra-articular, topical, and aerosols for inhalation (9).

These drugs are also classified according to the duration of their action. Short-acting corticosteroids contain cortisone and cortisol (hydrocortisone), with an action time of less than 12 hours and an anti-inflammatory potency of one. Intermediate-acting has an action time between 12 and 36 hours; these contain prednisone and prednisolone with an anti-inflammatory potency of 4, and 6-methylprednisolone and triamcinolone have a potency of 5 and are examples of intermediate-acting drugs. Betamethasone and dexamethasone are long-acting glucocorticoids with a time of action greater than 36 hours and a potency of the anti-inflammatory character of 25 hours (10). Methylprednisolone and dexamethasone are the most often corticosteroids used after impacted third molar surgery to decrease the initial inflammatory response. These can be administered by injection into the surgical area or systemically (10,11).

Another previous systematic study (11) made the comparison between both drugs (methylprednisolone and dexamethasone); therefore, it restricted the study to those medications only. Then, the primary goal of this systematic study was to evaluate the use of methylprednisolone in the postoperative period of impacted third molars in relation to its efficacy in postoperative pain and edema, dosage regimens, forms of administration, and adverse effects. Secondarily, the aim was to analyze the impact of methylprednisolone after the extraction of impacted teeth compared to no medication use or the use of different drugs from the same pharmacotherapeutic group.

## Material and Methods

This systematic review followed the PRISMA (Preferred Reporting Items for Systematic Reviews and Meta-Analyses) statement (12). This study was registered in PROSPERO (CRD42024512561). The PICO (Population/Intervention/Comparison/Outcome) strategy was used to formulate the clinical focus question: “In patients who underwent impacted third molar extraction (P), what was the effect of methylprednisolone used postoperatively (I) compared to non-use or the use of other medications within the same pharmacotherapeutic group (C) to reduce inflammation and pain (O)?” The outcomes observed were: (a) reduction of postoperative pain, (b) postoperative edema, (c) other complications of inflammatory origin, (d) trismus, (e) need for parallel analgesia, and (f) quality of life after extraction.

- Eligibility Criteria

The inclusion criteria were: 1. Randomized controlled trials (RCTs), 2. published within less than 10 years (2013-2023), 3. with a minimum of 10 patients, 4. published in English, and 5. reported the use of methylprednisolone in the postoperative period of extraction of impacted teeth. The exclusion were: 1. controlled clinical trials (CCTs), clinical studies, reviews, *in vivo*, *in vitro* studies, case series, and case reports, 2. studies that ignored the chronic use of medications that could interfere with the results, 3. studies that included patients with non-controllable systemic disease, 4. studies that included smokers, and 5. studies with a lack of information or detail and missing follow-up descriptions.

- Search strategy and Data extraction

Two independent investigators (HL and BL-A) performed an electronic search in the following databases: PubMed/MEDLINE and Scopus to find articles about methylprednisolone that were related to the extraction of third molars. A manual search was also performed. The combination of specific keywords was applied in each database, associated with Boolean operators: 1. PubMed/MEDLINE: (methylprednisolone) OR (corticosteroids) OR (steroids) AND ((third molar) OR (impacted teeth) OR (impacted tooth)); it was applied filters to adjust the result; 2. Scopus: (methylprednisolone) OR (corticosteroids) OR (steroids) AND ((third molar) OR (impacted teeth) OR (impacted tooth)); with filters: Limited to dentistry/limited article, clinical studies, RCT, CCT, Review, English, and 2013-2023. The agreeability inter-reviewer was assessed using Cohen’s Kappa test.

The data were extracted based on the general study design, year of publication, type of study, number of patients included, gender, age, follow-up, detail of the surgeries, dosage of the medication, drug administration route, method of swelling, maximum mouth opening (MMO) evaluation, and pain assessment.

- Quality assessment and Statistical analysis

Two reviewers (HL and BL-A) independently assessed the quality of the study; in case of disagreement, a third author (TB) was consulted. The risk of bias for the RCTs included was performed using a revised Cochrane risk-of-bias tool for randomized trials (RoB2). The following domains were observed: the randomization process, deviations from intended interventions, missing outcome data, measurement of the outcome, and selection of reported outcomes. The low risk of bias was represented by the green color and indicated that the study implemented robust measures, minimizing the risk of bias in the respective domain; uncertain risk of bias (yellow) suggested that the study lacks clear information or details, making it difficult to determine the risk of bias; high risk (red) showed the study had significant limitations or flaws in the design or execution, increasing the risk of bias. Suppose all parameters were filled with low risk (green) or until two unclear (yellow), the overall result was a low risk of bias (green). For results with only one high risk (red) and up to two unclear (yellow), the result was a moderate risk of bias. Whereas if filled with 2 or more high risks (red) and/or more than 2 unclear risks present (yellow), the overall result will be a high risk of bias (13).

The random effect model was used for the meta-analysis to evaluate the variables. Heterogeneity was analyzed using Cochran's Q test and Higgins' I2 statistics. Standardized mean differences were used to measure the effect. The statistical analyses were carried out using Review Manager 5.4 software. A comparative analysis of the studies' results was used to analyze variables where meta-analysis was not possible.

## Results

**Figure 1:**
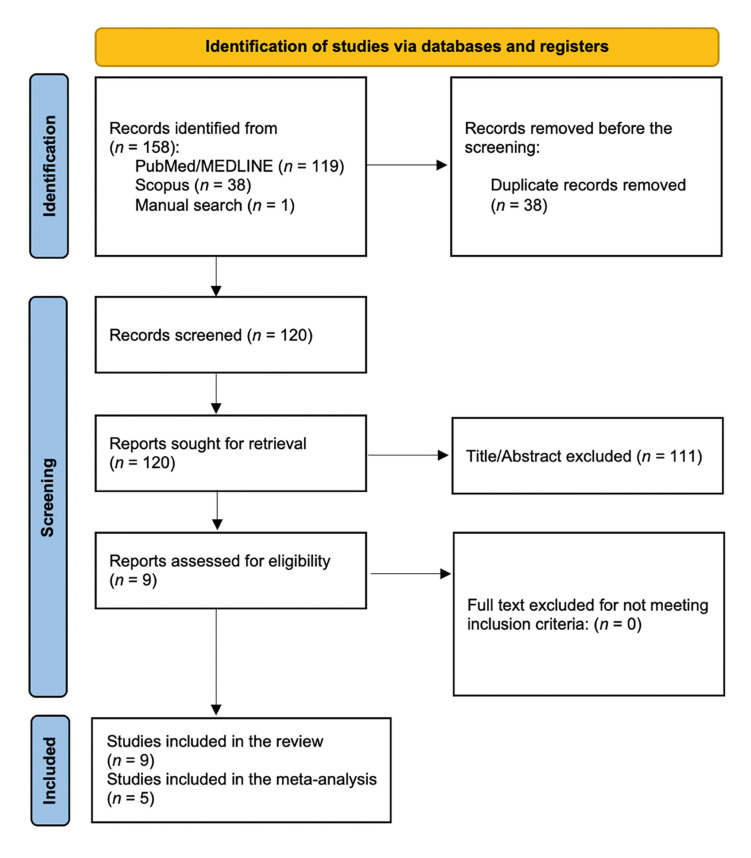
PRISMA flowchart for the selection and inclusion of the studies.

The search queries identified 158 studies; 149 were excluded due to duplicates and/or did not meet the predefined eligibility criteria. These were excluded after the initial evaluation of the title and abstract. Nine articles were included for full-text analysis, resulting in the nine articles included (14-22) in this systematic review that met the eligibility criteria (k=0.92) (Fig. 1).

- Study characteristics and General assessment

The data was collected and exposed in Table 1. The 9 RCTs included evaluated 354 patients, with a mean age of 25.53 years; 475 surgeries were developed. Except for Chugh *et al*.’s (16) and Selvaraj *et al*.’s (22) studies, female patients prevailed. The route of drug administration varied through oral, muscle, submucosal, or intravenous (Table 1). There was great variability in sample size, with one study having a sample of 10 patients and another with 65 patients, giving a mean value of 39.33 ± 21.18 patients per study. The average age of the patients is relatively close between the studies, except for Alcantara *et al*.’s (14) study, which has the lowest average value, 20.3, and Koçer *et al*.’s (18) study, which has the highest average value, 29.6. The number of surgeries reported in the studies varied between 20 and 104 (mean of 52.78 ± 24.06), which reveals a disparity in the number of surgeries analyzed in the studies.

Five studies (15,16,18,19,21) compared methylprednisolone and placebo results in the postoperative period after impacted third molars extraction. They assessed the postoperative trismus, examined the patients' range of mouth opening after impacted third molar extraction surgery, and noted any restriction in mandibular movement (subjective appraisal). In addition, those authors also evaluated the postoperative pain, measuring the intensity and duration reported. Standard pain assessment scales were used, allowing a more in-depth understanding of the effects of methylprednisolone on postoperative pain relief compared to the control/placebo group. Furthermore, the same studies also evaluated postoperative swelling, which permitted observing the facial swelling levels after extraction. The soft tissues around the surgical region were examined, noting any noticeable increase.

Five studies (14-16,20,22) compared methylprednisolone and dexamethasone in the postoperative period. Postoperative pain was assessed exclusively using the Visual Analog Scale (VAS); edema was evaluated through the presence and magnitude of its dimension; and trismus was measured through the mouth opening amplitude. These measurements were carried out using standardized assessment instruments, recording the distance between anatomical reference points in the mandible and maxilla. This assessment method allowed a quantitative analysis of trismus, providing objective data on the extent of mouth opening limitation following impacted third molar extraction.

Regarding the routes of administration for methylprednisolone in the postoperative period, three studies (23,24,27) performed the comparison. Koçer *et al*. (18) evaluated the oral, intravenous, and masseter routes; and Gholami *et al*. (19) and Selvaraj *et al*. (22) investigated the gluteal and masseter muscle routes. This comprehensive analysis of these studies aims to examine the relative efficacy of methylprednisolone administered by different vias, offering valuable insights for clinical practice by identifying the most effective route of administration to optimize the control of postoperative pain, edema, and trismus.

- Clinical outcomes

Table 2 shows the details of the parameters analyzed. Table 3 summarizes the inter-incisal reduction/MMO and shows the outcomes obtained comparing methylprednisolone and the control group. Koçer *et al*.’s (18) and Chugh's (16) results show that the experimental group had less reduction and decreased over time. On the other hand, in the 7-day results of Chugh *et al*. (16) and Darawade *et al*. (20), the average reduction was higher in the experimental group. Table 3 summarizes the studies that reported results on inter-incisal reduction using only methylprednisolone (single group).

Comparing methylprednisolone and dexamethasone (Table 4), the pain increased over time in both groups in the Alcantara *et al*. study (14); in the Srivastava *et al*. (17) and Darawade *et al*. (20) studies, it decreased in the methylprednisolone group and remained the same in the dexamethasone group over time.

For the swelling analysis, Table 4 show the results for Tragus-Commissure and Canthus-Gnathion edema, respectively, reported in Koçer *et al*.'s study (18). For Tragus-Commissure, the experimental group has lower average values than the control group in all three studies, and in both groups, the results decrease over time. For Canthus-Gnathion, the experimental group showed higher results on average than the control group in all three studies; in both groups, the results decreased over time.

- Quality assessment and Statistical analysis

**Figure 2:**
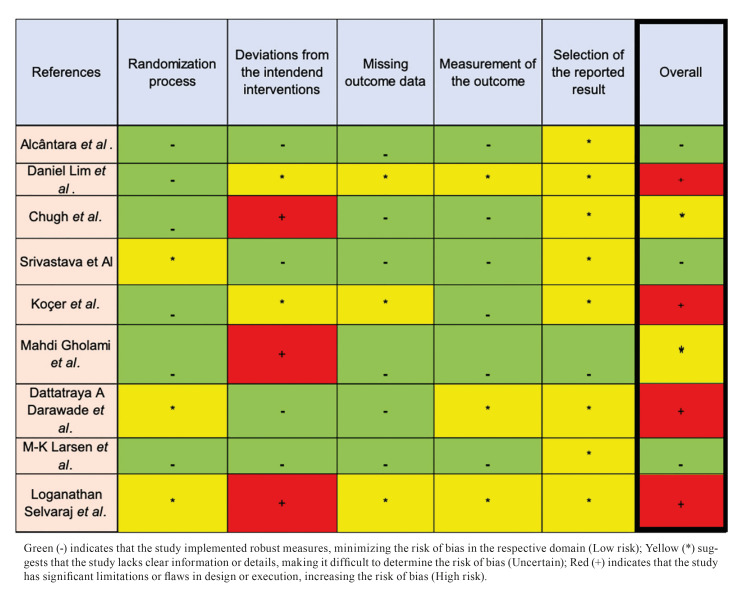
Overall Risk of Bias Assessment using RoB2 tool.

Fig. 2 presents the risk of bias for the included RCTs. Three studies had a low risk of bias, 2 had a moderate risk, and 4 had a high risk. Due to limitations in the included literature, the meta-analysis was only done for specific parameters. The careful selection of parameters was based on the availability and comparability of data, guaranteeing the robustness and validity of the results obtained.

**Figure 3:**
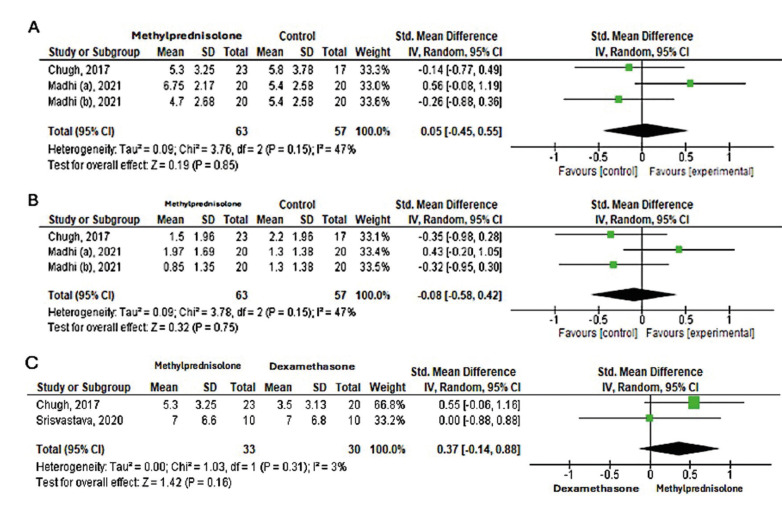
Meta-analysis results for pain after (A) 24h and (B) 7 days comparing methylprednisolone with placebo, and (C) after 24h comparing methylprednisolone with dexamethasone.

Fig. 3 shows the pain results after 24 hours and 7 days, comparing the result of methylprednisolone with placebo (control). After 24 hours, Cochran's Q (*p*=0.15>0.05) and I2=47%, it was observed moderate heterogeneity; the forest plot shows that the effect of the meta-analysis 0.05 (95%CI [-0.45;0.55]) was not statistically significant (*p*=0.85). After 7 days, the forest plot showed the effect of the meta-analysis -0.08 (95%CI [-0.58;0.42]), which was also not statistically significant (*p*=0.85). Analyzing the data for the comparison between methylprednisolone and dexamethasone, Cochran's Q (*p*=0.31>0.05) and I2=3%, it was concluded there was homogeneity between studies after 24 hours; the forest plot shows that the effect of the meta-analysis 0.37 (95%CI [-0.14;0.88]) was not statistically significant (*p*=0.16).

**Figure 4:**
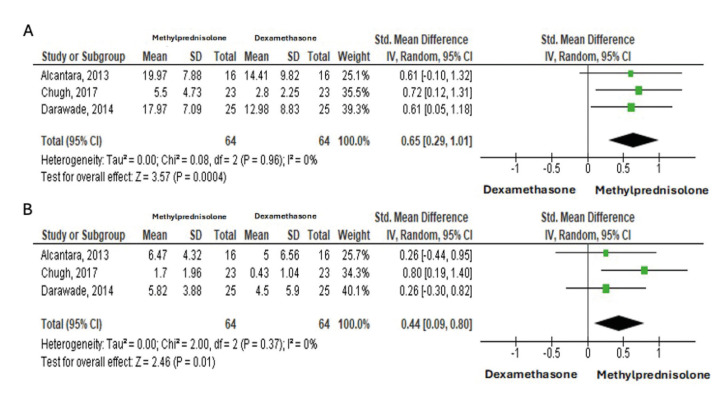
Meta-analysis results for inter-incisal reduction after 48 hours (A) and after 7 days (B).

Fig. 4 shows the inter-incisal/MMO reduction results at 48 hours and 7 days. Given the results, Cochran's Q (*p*=0.96>0.05) and I2=0% (A) and Q (*p*=0.37>0.05) and I2=0% (B), it was concluded that there was homogeneity between studies about inter-incisal/MMO reduction at 48 hours and 7 days. The forest plot presented the most significant reduction occurred in the methylprednisolone group, and the effect was 0.65 (95%CI [0.29;1.01]) (A) and 0.44 (95%CI [0.09;0.80]) (B), respectively, statistically significant result (*p*<0.01 and *p*<0.05).

## Discussion

The aim of this systematic review was to assess the effect of methylprednisolone in patients undergoing third molar surgery, with a specific focus on post-operative trismus, pain, and edema. This analysis was carried out considering the predefined criteria, grouping all available randomized controlled trials (RCTs) that investigated the use of methylprednisolone in comparison with a control group, as well as in comparison with dexamethasone.

For the dosage of corticosteroids to be effective, it must exceed the physiological production of the human body (5-30mg/day) (23). The doses used in this review were 20 mg and 40 mg. The results of this review showed that methylprednisolone was efficient in reducing pain, edema, and trismus. It is similarly observed in the literature, where the corticosteroids, e.g., dexamethasone and methylprednisolone, showed the ability to inhibit the immune system. It is an anti-inflammatory with analgesic and anti-allergic potential. Moreover, the toxicology proved that both are safe at low dosages via oral or other use routes (24). Therefore, careful instructions should be passed to the patients when using glucocorticoids.

The literature has shown that methylprednisolone, at standard doses, was considered more effective than dexamethasone and prednisolone for treatments (25) and five times more potent than hydrocortisone (26). It suggests that methylation of prednisolone makes it more potent by aiding interaction with a cellular target rather than increasing its stability. Otherwise, the long-term use of these medications and when used in high dosages, may cause some undesired side effects (steroid osteoporosis, steroid diabetes, delayed wound healing, and are closely associated with mortality) (27). Methylprednisolone exhibited linear plasma protein binding (average of 77%) and was moderately distributed into tissue spaces; however, the volume of distribution of prednisolone is only one-half that of methylprednisolone, proving the superiority of this drug (28).

Within this context, the results of the present review revealed after 48 hours and 7 days that dexamethasone had lower inter-incisal/MMO reduction compared to methylprednisolone (respectively, *p*<0.01 and *p*<0.05). Agrawal *et al*.'s (29) results agree with the evidence presented herein, reinforcing the efficacy of corticosteroids. This consistency strengthens the evidence of the positive potential of corticosteroids in mitigating postoperative trismus, providing additional support for their effectiveness in promoting a more comforTable and effective recovery after third molar surgery (30).

Regarding pain, after 24 hours, there was no significant effect between methylprednisolone and dexamethasone (*p*=0.16). This highlights the complexity of post-operative pain, which is influenced by different factors such as surgical trauma and the individual inflammatory response and threshold. Variability in pain administration and measurement methods between studies may also have contributed to these results. Furthermore, the prophylactic oral intake of dexamethasone 8 mg was compared to 4 mg to verify the control of the postsurgical edema after third molar extractions; 8 mg presented greater efficacy in the control of postsurgical edema (31). The literature also suggests other medications, such as the postoperative administration of 30 mg prednisolone; it demonstrated relief for trismus, swelling, and pain compared to non-steroidal anti-inflammatory (NSAI) (control group); this fact shows the potential of prednisolone. There was a significant reduction of swelling (*p*<0.05) and higher MMO and visual analogue scale (VAS) for the use of prednisolone, without any disturbance of wound healing or other corticosteroid-related complications (32). In addition, another medication typically prescribed is ibuprofen (NSAI). Ibuprofen and methylprednisolone are the two drugs largely used in this type of surgery (third molar extraction) (2); Schultze-Mosgau *et al*. (33) showed that the combination of both medications allowed to obtain better analgesic and anti-inflammatory effects than the separate use. López Carriches *et al*. compared methylprednisolone and diclofenac in reducing trismus (34). Similarly, Bamgbose *et al*. also verified the absence of differences in the control of trismus when they compared a group medicated with dexamethasone and diclofenac versus using only diclofenac (35). On the other hand, the results obtained by Troullos *et al*. supported the existence of differences between the tested groups, with considerably lower trismus values ​​in patients medicated with methylprednisolone compared to those medicated with ibuprofen (36).

Comparing the methylprednisolone with the control groups included, the pain parameter was observed after 24 hours and 7 days; in both periods, no significant effect was observed in favor of methylprednisolone (*p*=0.85). This suggests that methylprednisolone may not be more effective than the control assessed in reducing pain at these time intervals. However, it is essential to consider that postoperative pain is a subjective experience and can be influenced by various factors (such as the type of surgery performed, the patient's psychological state, the presence of pre-existing conditions, the quality of pain management, and the level of social support) (37). Subgroup analyses may provide additional insights into the efficacy of methylprednisolone in different populations and surgical contexts (18). Another study (38) also reported no significant differences in pain between the groups treated with methylprednisolone and the control groups at the same period. These results highlight the need to consider postoperative pain as a multifaceted experience influenced by a variety of factors and the importance of more detailed analyses to understand the effects of methylprednisolone on postoperative pain management fully.

In 2023, a systematic study (11) recently compared methylprednisolone versus dexamethasone's efficacy in managing post-surgical pain, swelling, and trismus after surgery. Some methodological differences were observed compared to our study: 1. the present systematic study compared the results of methylprednisolone with any other medication or no medication, while the other systematic included articles that performed only the comparison between dexamethasone versus methylprednisolone; 2. the authors included other databases besides what was used in the present study, which can obtain a different inclusion; 3. the authors considered articles published without publication date or language restrictions, whereas the present study included some limitations (articles published in the last 10 years [2013-2023] and English language); and 4. there was differences in the eligibility criteria comparing both systematic studies. Otherwise, a similar result was found for dexamethasone, which had statistically significant and better results than methylprednisolone in reducing trismus.

- Limitations of the Study

As a limitation, it is possible to verify that a low number of RCTs have been developed on the subject. Also, many of the articles needed to have more crucial values. Several studies did not report complete measurements, and many did not include standard deviations, which made it impossible to include these studies in the meta-analysis. In addition, the divergence in the measures used between the different studies represented a significant challenge. For example, when assessing trismus, we opted to use the inter-incisal reduction measure since most of the available articles employed this specific method, providing greater consistency and comparability between studies. However, when trying to convert data from articles that measured maximum mouth opening to the inter-incisal reduction measure, we often lost the standard deviation value, making it impossible to calculate this crucial statistic for the meta-analysis accurately. Similarly, there was significant variability in the measurement methods used between the different studies in assessing edema. This methodological heterogeneity and the lack of reported standard deviations made it difficult to harmonize the data, limiting the possibility of carrying out a comprehensive and comparative meta-analysis.

- Final considerations

Within the limitations of this study, it was possible to verify that methylprednisolone efficiently treats patients safely after third molar extraction, reducing pain, edema, and trismus. There was a positive trend for edema reduction via masseter muscle; however, there was no significant difference or comparable data available regarding pain, the best route of administration, adverse effects, and patient post-operative comfort. Methylprednisolone achieved better inter-incisal level/MMO results than dexamethasone; otherwise, dexamethasone is preferable in minimizing postoperative trismus, presenting superior potential in this specific clinical context. All data should be carefully analyzed due to the study's limitations, the small number of studies included, heterogeneity found, and the risk of bias present. More RCT studies are recommended to confirm the findings of this study.

## Figures and Tables

**Table 1:** Demographic and study design information.

Author	Year	Type of study	nº of patients	Gender	Mean age	Follow-up	nº of surgeries	Dosage of administration (route)
Alcântara *et al.* (13)	2014	RCT	16	3M/12F	20.3 ±1.25	24h/48h/72h/7 days	32	Group 1: 8mg Dexamethasone (oral) Group 2: 40mg Methylprednisolone (oral)
Lim *et al.* (14)	2017	RCT	65	11M/49FGroup 1: 3M/17F Group 2: 0M/20F Group 3: 8M/12F	Mean: 25±4 Group 1: 24.2 Group 2: 25.2 Group 3: 25.8	24h/48h/72h/(5/7) days	65	Group 1: Control (placebo) Group 2: 4mg Dexamethasone (masseter muscle) Group 3: 40mg Methylprednisolone (masseter muscle)
Chugh *et al.* (15)	2017	RCT	60	38M/22F	29.7	48h/7 days	60	Group 1: Control (saline solution) Group 2: 8mg Dexamethasone (submucosal) Group 3: 40mg Methylprednisolone (submucosal)
Srivastava *et al.* (16)	2020	RCT	20	7M/13F	26.7	24h/48h/72h/(4/5/7)days	40	Group 1: 40mg Methylprednisolone (masseter muscle) Group 2: 8mg Dexamethasone (masseter muscle)
Koçer *et al.* (17)	2014	RCT	44	18M/26F	29.6	48h/7 days	44	Group 1: Control Group 2: 20mg Methylprednisolone (masseter muscle) Group 3: 20mg Methylprednisolone (oral) Group 4: 20mg Methylprednisolone (intravenous)
Gholami *et al.* (18)	2021	RCT	60	29M/31FGroup 1: 8M/12F Group 2: 13M/7F Group 3: 8M/12F	Mean: 27.55 Group 1: 27.55 Group 2: 27.25 Group 3: 25.7	24h (5/7) days	60	Group 1: 40mg Methylprednisolone (masseter muscle) Group 2: 40mg Methylprednisolone (gluteal muscle) Group 3: Control
Darawade *et al.* (19)	2014	RCT	25	NR	25.9±6	24h/48h/72h/7 days	50	Group 1: 8mg Dexamethasone (oral) Group 2: 40mg Methylprednisolone (oral)
Larsen *et al.* (20)	2021	RCT	52	16M/36F	27±6	24h/72h/7days/1 month	104	Group 1: 20mg Methylprednisolone (masseter muscle) Group 2: 30mg Methylprednisolone (masseter muscle) Group 3: 40mg Methylprednisolone (masseter muscle) Group 4: Saline solution
Selvaraj *et al.* (21)	2014	RCT	10	6M/4F	27±6	48h/7 days	20	Group 1: 40mg Methylprednisolone (masseter muscle) Group 2: 40mg Methylprednisolone (gluteal muscle)

NR: Not Reported; F: female; M: male.

**Table 2:** Clinical outcomes design.

Author	Swelling assessment	MMO	Pain assessment
Alcântara *et al.* (13)	Sum of the three measurements (corner of the eye/angle of the mandible-tragus/corner of the mouth-tragus/pogonion)	Reduction in MMO from baseline value	VAS score (0-10)
Lim et al. (14)	Sum of 2 lines-corner of the eye to angle and tragus to corner of mouth. Measure as % change in baseline value. Reported in graphical data only	Maximum interincisal distance between pre-operative and post-operative days	VAS score (0-10)
Chugh et al. (15)	Sum of 2 lines-corner of the eye to angle and tragus to corner of mouth. Measure as mean change in base value in mm	Reduction in MMO from baseline value	VAS score (0-10)
Srivastava et al. (16)	Lines: Tragus-Commissure + Gonion-external canthus + gonion-commissure	Reduction in MMO from baseline value	VAS score (0-10)
Koçer et al. (17)	Lines: Canthus-gnathion line; tragus-commissure line; tragus-pogonion	The distance between the upper and lower incisal borders of the central incisors was measured using a digital caliper	NR
Gholami et al. (18)	Measured by ultrasound imaging	Maximum interincisal distance between pre-operative and post-operative days	VAS score (0-10)
Darawade et al. (19)	Tape measuring method described by Ustun et al.	Measure of the distance between the right upper and lower central incisors with the help of a caliper	VAS score (0-10)
Larsen et al. (20)	NR	Maximum interincisal distance between pre-operative and post-operative days	VAS score (0-100)
Selvaraj et al. (21)	Distance between tragus-lip commissure, gonion-lip commissure and gonion-external canthus of the eye using surgical silk thread	Measured with Caprovich callipers	VAS score (0-100)

MMO: Maximum mouth opening; NR: Not Reported.

**Table 3:** Summary of studies on inter-incisal reduction.

	Author	Administration route	Dose (mg)	Control (mm)			Methylprednisolone (mm)		
				Average (48h)	Average (7d)	nº	Average (48h)	Average (7d)	nº
MMO	Koçer *et al.* (a)	Oral	20	17.5	11.1	11	13.3	5.1	11
	Koçer *et al.*(b)	IV	20	17.5	11.1	11	8	1.7	11
	Koçer *et al.* (c)	Muscular (masseter)	20	17.5	11.1	11	7.7	1.6	11
	Chugh *et al.*	Submucosal	40	7.2±4.54	1.4±2.45	17	5.5±4.73	1.7±3.09	23
	Darawade *et al.*(a)	Muscular (masseter)	40	NR	3.55	20	NR	8	20
	Darawade *et al.*(b)	Muscular (gluteal)	40	NR	3.55	20	NR	6.33	20
MMO (Methylprednisolone - single group)	Selvaraj *et al.*(a)	Muscular (masseter)	40	-	-	-	NR	10.8	4.7
	Selvaraj *et al.* (b)	Muscular (gluteal)	40	-	-	-	NR	11.1	3.3
	Alcantara *et al.*	Oral	40	-	-	-	16.27±8.13	19.97±7.88	6.47±4.32
	Lim *et al.*	Submucosal	40	-	-	-	8	7	2
	Srisvastava *et al.*	Muscular (masseter)	40	-	-	-	NR	15.85	7.15
	Darawade *et al.*	Oral	40	-	-	-	14.64±7.30	17.97±7.09	5.82±3.88

NR: Not reported; IV: intravenous; MMO: Maximum mouth opening; h: hours; d: days.

**Table 4:** Overview and summary of pain, edema (Tragus-Commissure), and edema (Canthus-Gnathion).

	Author	Administration route	Dose (mg)	Dexamethasone (mm) - Pain Control (mm) - Edema				Methylprednisolone (mm)			
				Preop.	Average (1st period)	Average (2nd period)	nº	Preop.	Average (1st period)	Average (2nd period)	nº
Pain (1^st^ period: 24h/ 2^nd^ period: 48h)	Alcantara *et al.*	Oral	40	-	2	4	16	-	1	2	16
	Srivastava *et al.*	Muscular (masseter)	40	-	7±6.60	6±6.05	10	-	7±6.80	7±6.45	10
	Chugh *et al.*	Submucosal	40	-	5.3±3.25	NR	23	-	3.5±3.13	NR	20
	Darawade *et al.*	Oral	40	-	2.5	1.5	25	-	1.5	1.5	25
Edema (Tragus-Commissure) (1^st^ period: 48h/ 2^nd^ period: 7d)	Koçer *et al.* (a)	Oral	20	114	126.4	120.6	11	114	123.0	117.5	11
	Koçer *et al.* (b)	IV	20	114	126.4	120.6	11	116.6	122.4	117.3	11
	Koçer *et al.* (c)	Muscular (masseter)	20	114	126.4	120.6	11	111.2	114.6	111.6	11
Edema (Canthus-Gnathion) (1^st^ period: 48h/ 2^nd^ period: 7d)	Koçer *et al.*(a)	Oral	20	92	98.3	95.1	11	99.6	103.6	100.5	11
	Koçer *et al.*(b)	IV	20	92	98.3	95.1	11	109.6	112.8	110.3	11
	Koçer *et al.* (c)	Muscular (masseter)	20	92	98.3	95.1	11	101.9	102.9	101.9	11

NR: Not reported; Preop.: preoperatively; IV: intravenous; d: days; h: hours; mm: millimeters.
